# Cytotoxic Effect of Phenylethanoid Glycosides Isolated from *Plantago lanceolata* L.

**DOI:** 10.3390/life13020556

**Published:** 2023-02-16

**Authors:** Anna Budzianowska, Ewa Totoń, Aleksandra Romaniuk-Drapała, Małgorzata Kikowska, Jaromir Budzianowski

**Affiliations:** 1 Laboratory of Pharmaceutical Biology and Biotechnology, Department and Division of Practical Cosmetology and Skin Diseases Prophylaxis, Poznan University of Medical Sciences, 60-806 Poznań, Poland; 2 Department of Clinical Chemistry and Molecular Diagnostics, Poznan University of Medical Sciences, 60-806 Poznań, Poland

**Keywords:** ribwort plantain, plantamajoside, acteoside, isorhamnetin triglycoside, MTT test, cytotoxicity, cancer cell lines

## Abstract

The aim of the study is to investigate whether the bioactive compounds isolated from *P. lanceolata* inflorescences, namely, phenylethanoid glucosides, acteoside, plantamajoside, and a flavonoid, isorhamnetin-3-*O*-rutinoside-4′-*O*-glucoside, possessed cytotoxic activity against the selected cancer cell lines. The potential antitumor effects of two phenylethanoid glycosides and one flavonoid were evaluated via MTT (3-(4,5-dimethylthiazol-2-yl)-2,5-diphenyltetrazolium bromide) assay on seven human carcinoma cell lines (MCF-7, MDA-MB-231, Caco-2, HepG2, OVCAR-3, U138-MG, U251-MG) and one nontumorigenic mammary epithelial cell line (MCF-12A). For the first time, acteoside was studied in ovarian cancer cell line OVCAR-3, and plantamajoside in all cell lines except breast adenocarcinoma MDA-MB-281 and hepatocarcinoma HepG2. The phenylethanoid glycosides showed stronger cytotoxic activity than that of the glycoside flavonoid. Acteoside and plantamajoside, at concentrations of 200 and 300 μM, respectively, had a highly toxic effect on the selected two cancer cell lines of breast adenocarcinoma MDA-MB-231 and MCF-7, ovarian cancer cell line OVCAR-3, glioblastoma cell line U138-MG, and hepatocarcinoma cell line HepG2. Both glycosides were significantly less cytotoxic towards nontumorigenic cell line MCF-12A; the effect appeared at a concentration of 400 μM. For the first time, the activity of acteoside and plantamajoside was compared in one parallel investigation. The results are discussed against a broad background of existing knowledge on biological effects, their mechanisms, and structure–activity relationships. Phenylethanoids may be potential compounds with cytotoxic activity against the selected cancer types.

## 1. Introduction

*Plantago lanceolata* L. (ribwort plantain), belonging to the Plantaginaceae family, is a perennial species with a global distribution, including the flora of Europe [1,2]. *P. lanceolata*, a well-known medicinal plant, has antispasmodic, anti-inflammatory, antioxidant, antibacterial, antiasthmatic, and cytotoxic activities [3,4,5,6,7,8]. It contains mainly iridoids and phenylethanoid glycosides [9,10,11] with biologically active compounds, especially aucubin [12], acteoside [13] and plantamajoside [14]. Flavonoids were also identified, mainly apigenin and luteolin glucuronides [15,16], and a few flavonol, 3-*O*-glycoside, at a much lower content than that of flavones [17,18]. The medicinal material approved in official monographs, and named *Plantaginis lanceolatae folium*, is defined as leaves and scapes [19,20], leaves, or flowering aerial parts (*Plantaginis lanceolatae herba*) [21].

**Figure 1:**
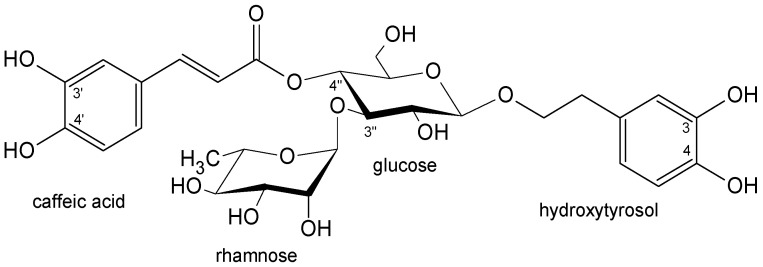
The chemical structure of acteoside.

Acteoside (also known as verbascoside or kusaginin), a natural phenylethanoid glycoside, is derived from hydroxytyrosol and *trans*-caffeic acid, both linked to glucose, which is further substituted with rhamnose (Figure 1). It is a widely distributed compound in the plant kingdom that was detected in more than 200 plant species representing 23 plant families [13,22]. All scientific information regarding the compound demonstrated a wide range of biological activities, including antioxidant, anti-inflammatory, antiepileptic, neuroprotective, antifungal, antiviral, and anticancer [13,23,24]. 

**Figure 2:**
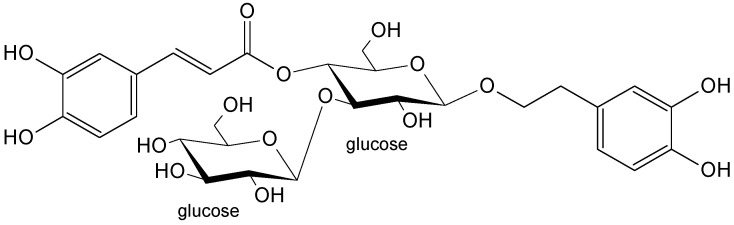
The chemical structure of plantamajoside.

Plantamajoside is a phenylethanoid glycoside that differs from acteoside by having glucose in the place of rhamnose (Figure 2). Only 33 plant species from 3 plant families, namely, Plantaginaceae, Gesneriaceae and Orobanchaceae, contain plantamajoside. This phenylethanoid occurs mainly in the Plantaginaceae family (30 species) and particularly in the *Plantago* genus (20 species) [14]. It is a rarer and less-studied compound that exhibits multiple biological and pharmacological properties: Anti-inflammatory, diuretic, wound-healing, antiasthmatic, hepatoprotective, antiaging, antibiotic, antifungal, enzyme-inhibitory and neuroprotective, and anticancer [14,25]. 

**Figure 3:**
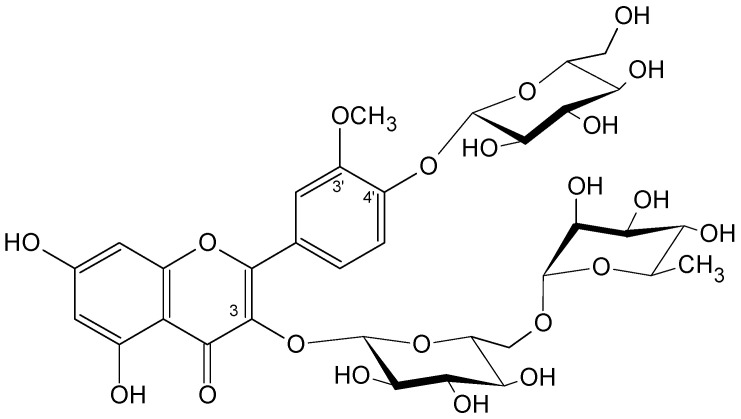
The chemical structure of isorhamnetin-3-*O*-rutinoside-4′-*O*-glucoside.

Isorhamnetin-3-*O*-rutinoside-4′-*O*-glucoside (Figure 3) was recently reported as a main flavonoid of the inflorescence of *Plantago lanceolata* and a new compound for the *Plantago* genus [26]. It was not investigated previously for biological activity, in contrast to its derivatives, isorhamnetin-3-*O*-rutinoside and isorhamnetin-3-*O*-glucoside, which exert anticancer properties [27].

According to the World Health Organization [28] and World Cancer Research Fund International [29], cancer is a major global public health issue and will become the most common major cause of death in the near future. The abnormal and uncontrollable growth of cells may start at any tissue or organ and spread to other parts of the body. In 2020, there were 2.3 million women diagnosed with breast cancer (685,000 deaths globally) and 313,000 new cases of ovarian cancer (the eighth most commonly occurring cancer in women). Colorectal (also known as bowel) cancer, with more than 1.9 million new cases in 2020, is the third most common cancer worldwide. Liver cancer is the sixth most common cancer worldwide, with more than 900,000 new cases in 2020. Glioblastoma is one of the most common and detrimental forms of solid brain tumors. Statistical data on cancer incidence and mortality are frightening. However, as oncologists argue, many types of cancer can be successfully cured if detected at an early stage and treated effectively. The development of novel anticancer drugs assumes avoidinig harmful effect on normal cells, which is often a huge challenge. New compounds that may act as chemotherapeutic drugs need to be discovered because not all tumors react in the same way to treatment. Numerous scientific units and research centers globally are faced with this problem in cancer treatment, starting with the initial research on isolated cancer cell lines in vitro [28,29]. Several natural active agents of plant origin have been applied to the treatment of different types of cancer, i.e., vincristine and its derivatives (vinblastine, anhydrovinblastine, and the semisynthetic derivatives vindesine, vinorelbine, and vinflunine), etoposide, camptothecin (also irinotecan and topotecan), podophyllotoxin, and paclitaxel and its semisynthetic derivatives docetaxel and cabazitaxel derived from 10-baccatin III or 10-deacetylbaccatin III [30]. There is a plethora of compounds under preclinical and clinical trials, i.e., resveratrol, curcumin, epigallocatechin-3-gallate, quercetin, rutin, betulinic acid, and artemisinin. The substances include a wide spectrum of groups, such as alkaloids, diterpenes, diterpenoquinone, purine-based compounds, lactonic sesquiterpene, and exert cytotoxic properties with many different mechanisms of action. They may be used alone or in combination with synthesised drugs. In many cases, the isolation of compounds from plant extracts is more efficient, and less time-consuming and expensive compared to their chemical synthesis [30,31,32,33].

The aim of the study was to investigate whether the bioactive compounds isolated from *P. lanceolata* inflorescences, acteoside, plantamajoside, and isorhamnetin-3-*O*-rutinoside-4′-*O*-glucoside, possessed cytotoxic activity against the selected cancer cell lines: breast adenocarcinoma (MCF-7 and MDA-MB-231), colorectal adenocarcinoma (Caco-2), hepatocarcinoma (HepG2), ovarian cancer (OVCAR-3), glioblastoma (U138-MG and U251-MG), and a nontumorigenic epithelial cell line (MCF-12A).

## 2. Materials and Methods

### 2.1. Plant Material and Isolated Compounds 

The inflorescences of *Plantago lanceolata* L. were collected in June, July, and September from the plants cultivated in the Botanical Garden of the Department of Medicinal Plants (later Department of Medicinal and Cosmetic Natural Products) of Poznan University of Medical Sciences. The inflorescences were cut off the stems and dried at room temperature. The samples were extracted with boiling methanol (×3), and the dried extracts were separated into dichloromethane and water fractions via solvent partitioning. Water fractions that showed the same composition after chromatography analysis were combined and partitioned between water and n-butanol. The latter fraction was first separated via column chromatography on polyamide with a sequential elution with water, water-2-butanone mixtures, and methanol. The column fractions containing mixtures of flavonoids and phenylethanoid glycosides were further separated with a combination of preparative TLC on polyamide or avicel and column chromatography on polyamide, Sephadex LH-20, Cosmosil C_18_-OPN, Toyopearl HW-40 as described in detail in [26]. The isolated acteoside, plantamajoside, and isorhamnetin-3-*O*-rutinoside-4′-*O*-glucoside were structurally determined with nuclear magnetic resonance (^1^H and ^13^C NMR) spectroscopy as described in [26]. The purity of the studied compounds was >98% via ^1^H NMR spectroscopy [26].

### 2.2. Cell Culture 

Seven human cancer cell lines and one nontumorigenic mammary epithelial cell line were used in this study. Six cell lines were purchased from the American Type Cell Culture Collection (ATCC, Manassas, VA, USA) and one from the European Collection of Authenticated Cell Cultures (ECACC, Merck, Germany). The two breast adenocarcinoma cell lines MCF-7 (ATCC^®^ HTB-22™) and MDA-MB-231 (ATCC^®^ HTB-26™) were maintained as a monolayer in a complete growing RPMI-1640 medium (Biowest, Nuaillé, France) supplemented with 10% of fetal bovine serum (FBS) (Sigma-Aldrich, St. Louis, MO, USA). Colorectal adenocarcinoma cell line Caco-2 (ATCC^®^ HTB-37™) was maintained in Eagle’s Minimum Essential Medium (EMEM) (ATCC^®^, Manassas, VA, USA) with 10% FBS addition. Hepatocarcinoma cell line HepG2 (ATCC^®^ HB-8065TM) was maintained in medium EMEM supplemented with antibiotic-free 10% FBS (Sigma-Aldrich, St. Louis, MO, USA). Ovarian cancer cell line OVCAR-3 (ATCC^®^ HTB-161™) was cultured in an RPMI-1640 medium (Biowest, Nuaillé, France) supplemented with insulin (10 µg/mL) and 20% FBS (Sigma-Aldrich, St. Louis, MO, USA). The two glioblastoma cell lines U138-MG (ATCC^®^ HTB-16™) and U251-MG (ECACC, Salisbury, UK, cat. no. 09063001) were grown in a complete growing medium EMEM (ATCC^®^, Manassas, VA, USA), supplemented with 10% FBS (Sigma-Aldrich, St. Louis, MO, USA). Epithelial cell line MCF-12A (ATCC^®^ CRL-10782™) was maintained in DMEM-F12 medium (Biowest, Nuaillé, France) supplemented with hydrocortisone (0.5 µg/mL), insulin (10 µg/mL), human epidermal growth factor (hEGF) (20 ng/mL), cholera toxin (0.1 µg/mL), and 5% fetal horse serum (all purchased from Sigma-Aldrich, St. Louis, MO, USA). All cell lines were grown near confluence in 100 × 15 mm cell culture Falcon^®^Petri dishes (Corning, Poland) at 37 °C in an atmosphere containing 5% (*v*/*v*) of CO_2_ at 95% (*v*/*v*) of relative humidity. The absence of mycoplasma was checked routinely using the Mycoplasma Stain Kit (Sigma-Aldrich, St. Louis, MO, USA).

### 2.3. Cell Proliferation Assay

The cytotoxicity of the studied compounds was assessed on a broad spectrum of all tested cell lines using an MTT (3-(4,5-dimethylthiazol-2-yl)-2,5-diphenyltetrazolium bromide) assay. Cells were plated at a density of 5 × 10^3^ in 100 μL of the total medium dedicated to the cell line in sterile 96-well plates and cultured overnight. Then, the cells were divided into different groups: a control group (solvent without tested compounds), and groups treated with acteoside, plantamajoside, and isorhamnetin-3-*O*-rutinoside-4′-*O*-glucoside in a concentration range of 0–500 µM. All compounds were dissolved in dimethyl sulfoxide (DMSO) with a final solvent concentration of 0.25% (*v*/*v*), which did not affect cell viability. Cells were exposed to the studied compounds for 24, 48, and 72 h of incubation, and an MTT solution was added to each well (5.0 mg/mL) (Sigma-Aldrich, St. Louis, MO, USA). The cells were incubated at 37 °C for 4 h, followed by the addition of 100 μL of a solubilization buffer (10% SDS in 0.01 M HCl). Lastly, absorbance at 570 nm was measured using a Microplate Reader Multiscan FC (Thermo Scientific, Waltham, MA, USA), with a reference wavelength of 630 nm. Three separate experiments were performed with three repeats for each concentration. The viability of the cells was calculated with Excel software (Microsoft, Redmond, WA, USA), while the IC_50_ values were calculated using CompuSyn software (available for a free download from www.combosyn.com accessed on 27 September 2022) [34]. 

### 2.4. Statistical Analysis

The obtained data are expressed as the mean ± SD of at least three separate experiments. All statistical analyses were carried out using GraphPad Prism (GraphPad Software, College Station, TX, USA). Differences were assessed for statistical significance using repeated-measures ANOVA. The threshold for significance was *p* < 0.05; the symbols *, #, ♦, °, •, ∇, and × were used for *p* < 0.05, and **, ##, ♦♦, °°, ••, ∇∇, and ×× for *p* < 0.01.

The data from IC_50_ were analyzed using one-way analysis of variance (ANOVA), and statistical significance was determined using Duncan’s post hoc test (*p*-value < 0.05). All the analyses were conducted employing STATISTICA v. 13 (StatSoft, Inc. 2015). Mean values within a row (capital letters) and a column (small letters) within the same letters were not significantly different.

## 3. Results

The bioactive compounds isolated from *P. lanceolata* inflorescences acteoside (Figure 1), plantamajoside (Figure 2), and isorhamnetin-3-*O*-rutinoside-4′-*O*-glucoside (Figure 3) were investigated against seven human carcinoma cell lines (MCF-7, MDA-MB-231, Caco-2, HepG2, OVCAR-3, U138-MG, U251-MG) and one immortalized nontumorigenic mammary epithelial cell line (MCF-12A). A well-designed cytotoxicity assay should include a noncancer cell line to check for specificity. The MCF-12A cell line was developed from breast tissue with a nonmalignant fibrocystic disease. It was chosen as the control since it is nontumorigenic.

The cell viability of the studied compounds was assessed after 24, 48, and 72 h of treatment within the range of concentrations of 0–500 µM using an MTT assay. This wide range of concentrations was chosen on the basis of a variety of IC_50_ values reported for different cancer cell lines and the different effects of compounds observed at different concentrations [23].

**Figure 4:**
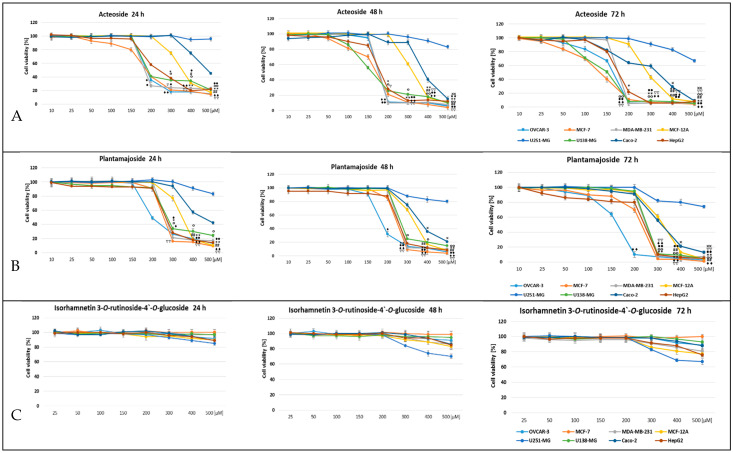
Viability assessment of OVCAR-3, MCF-7, MDA-MB-231, MCF-12A, U251-MG, U138-MG, Caco-2, and HepG2 cells after treatment with (**A**) acteoside, (**B**) plantamajoside, and (**C**) isorhamnetin 3-*O*-rutinoside-4′-*O*-glucoside for 24, 48, and 72 h. The cytotoxicity of the tested compounds was assessed using MTT proliferation assay as described in the Materials and Methods section. Cell viability is expressed as a percentage of the nontreated control cells. The final concentration of the solvent (DMSO) was 0.125%. The mean of three experiments ± SD is shown. Symbols *, #, ♦, °, •, ∇, × are for *p* < 0.05; **, ##, ♦♦, °°, ••, ∇∇, ×× for *p* < 0.01.

As the present study shows (Figure 4), the activity of the tested compounds was different depending on the compound’s chemical structure; phenylethanoid glycosides showed stronger cytotoxic activity than that of flavonoid glycoside. The cytotoxic effect was dose- and time-dependent in each case (Figure 4). Acteoside, at a concentration of 200 μM, had a highly toxic effect after 24 h of treatment on the selected two cancer cell lines of breast adenocarcinoma MDA-MB-231 and MCF-7, ovarian cancer cell line OVCAR-3, glioblastoma cell line U138-MG, and hepatocarcinoma cell line HepG2. Hepatocarcinoma cell line HepG2 and colorectal adenocarcinoma cell line Caco-2 were less sensitive to the tested compound, and the cytotoxic effect was induced at a higher concentration. No significant toxicity of the tested compounds was demonstrated in relation to glioblastoma cell line U251-MG. A similar structure of the plot of the cytotoxic effect of the tested compound concentration over time was observed for another phenylethanoid, plantamajoside. There was a clear cytotoxic effect of plantamajoside at a concentration of 200 μM on ovarian cancer cells, and at a concentration of 300 μM on other cancer cell lines, except U251-MG. Isorhamnetin 3-*O*-rutinoside-4′-*O*-glucoside did not show cytotoxic activity against any of the tested cancer lines regardless of the treatment time of the cells with this flavonoid up to a concentration of 200 μM; at a higher concentration, it showed very weak activity. Interestingly, in human mammary epithelial cell line MCF-12A, both compounds showed significantly lower cytotoxicity compared to that in breast cancer cell lines.

The lowest IC_50_ (the half maximal inhibitory concentration), and thus the highest activity of acteoside and plantamajoside were estimated for the cell lines of MCF-7, HepG2, OVCAR-3, U138-MG (Table 1). The lowest IC_50_ value was shown for acteoside in relation to the MCF-7 tumor and decreased over time, reaching 154.2 µM in 48 h and 113.1 µM in 72 h. The lowest IC_50_ value for plantamajoside was achieved against the HepG2 line and decreased over time, reaching 228.2 µM in 48 h and 156.1 µM in 72 h. The most interesting result was the very large discrepancy between the responses of the two glioblastoma cell lines, U138-MG and U251-MG, to the tested compounds.

The highest cytotoxic effect of the tested phenylethanoid glycosides (Table 1) was observed against the MCF-7 line. Both acteoside and plantamajoside showed IC_50_ values below 200 µM in this study: Acteoside: 154.2 µM after 48 h and 113.1 µM after 72 h; plantamajoside: 170.8 µM after 72 h. Most of the results were statistically different, and two had no difference. The obtained IC_50_ result of acteoside in relation to this line after 72 h was also the best result of the entire experiment. IC_50_ results below 200 µM were also obtained for the OVCAR-3 line. In that case, plantamajoside showed better activity (IC_50_-138.9 µM) than that of acteoside (IC_50_-162.8 µM), and both results after 72 h were statistically different. Acteoside also showed similar cytotoxic activity against the U138-MG line IC_50_ = 201.9 µM after 48 h and 156.6 µM after 72 h. The latter result was not statistically different from that obtained with acteoside against the OVCAR-3 line at the same time. On the other hand, plantamajoside had a weaker cytotoxic effect against the U138-MG line, showing the lowest IC_50_ after 72 h—266.7 µM. The last line for which the cytotoxic activity of both tested phenylethanoids was below 200 µM was HepG2. Both acteoside and plantamajoside showed IC_50_ values below 200 µM only after 72 h: Acteoside, 173.8 µM; plantamajoside, 156.1 µM. The two results were statistically different, but the 48 h values for the two compounds were not statistically different. The activity of acteoside against the MDA-MB-231 line was higher than that of plantamajoside (IC_50_ after 72 h, 200.2 and 263.1 µM, respectively), and the values were statistically different. The potency of acteoside and plantamajoside against the MCF-12A line was similar (IC_50_ about 300 µM); the value after 48 h for both compounds was not statistically different. Among the six studies on cell lines above, acteoside showed a stronger cytotoxic effect on the MCF-7, U138-MG, MDA-MB-231 lines, and plantamajoside was more potent on the OVCAR-3 line, with the two compounds having comparable potency on the HepG2 and MCF-12A lines. However, both compounds showed weaker activity against the Caco-2 line (IC_50_ after 72 h, 280.3 µM for acteoside and 316.4 µM for plantamajoside) and very weak activity against the U251-MG line—the lowest IC_50_ value was 985.6 µM after 72 h for plantamajoside (Table 1).

## 4. Discussion

This paper shows the results of the cytotoxic activity of two representatives (acteoside and plantamajoside) of an important class of compounds, phenylethanoids, on tumor cell lines that represent cancers that are the most common causes of death. Since the compounds of plant origin can be successfully applied to the treatment of different types of cancer, i.e., vinblastine, vincristine, etoposide, camptothecin, and taxol, there have been attempts to search for further compounds with such activity.

The anticancer effect of acteoside was confirmed in several previous earlier studies; e.g., cytotoxic activity was found on lymphocytic leukemia cells in P-388 mice (ED_50_ 2.6 µg/mL = 4.16 μM) [35]. Acteoside was cytotoxic to d R Lh-84 cells (rat liver cancer) (IC_50_ 61.98 µg/mL = 99.23 μM), HeLa (human epithelial carcinoma) (IC_50_ > 200 µg/mL = > 320 μM), S-180 (sarcoma) (IC_50_ 29.6 µg/mL = 47.39 μM), P-388/D1 (mouse lymphoid tumor) (IC_50_ 221.0 µg/mL = 353.83 μM), and HeLa cells (IC_50_ 7.8 µg/mL = 12.49 μM) [36]. Acteoside changed the malignancy of human gastric adenocarcinoma cell line MGc80-3 and induced its differentiation [37]. The acteoside-induced death of HL-60 promyelocytic leukemia cells was also observed (IC_50_ 26.7 µM) [38]. This phenylethanoid glycoside was also an inhibitor of protein kinase C (PKC), an enzyme released during cell proliferation and differentiation [39]. 

The cytotoxic activity of acteoside was recently studied by Şenol and coauthors (2021) on the MCF-7 and MDA-MB-231 cell lines and assessed using TEBU-BIO cell counting kit 8. Acteoside at 100 μM had the highest cytotoxic effect on MCF-7 breast cancer cells after 72 h of treatment. Moreover, the IC_50_ values for 24, 48, and 72 h acteoside exposure on MCF-7 cells were 0.127, 0.2174, and 0.2828 μM, respectively. In addition, 100 μM of acteoside had the highest cytotoxic effect on MDA-MB 231 cells after 24, 48, and 72 h of exposure. The IC_50_ values for 24, 48, and 72 h of acteoside exposure of MDA-MB 231 cells were 0.1597, 0.2584, and 0.2563 μM, respectively [40]. Due to the use of a different test and methodology, it is difficult to directly compare the results from this work and our experiment. Our research showed higher sensitivity of the MCF-7 cell line in comparison to the MDA-MB-231 response. These cell lines represent different molecular subtypes of breast cancer. MCF-7 cells (ER/PR+, HER2−, Ki-67−, TP53WT), in comparison to the MDA-MB-231 cells, are poorly aggressive and noninvasive. In contrast, the MDA-MB-231 basal-like subtype, also called triple-negative breast cancer (TNBC; ER/PR−, HER2, TP53mut), is a highly aggressive, invasive, and poorly differentiated cell line. Interestingly, in human breast epithelial cell line MCF-12A, acteoside showed significantly lower cytotoxicity compared to that in breast cancer cell lines. In this case, different responses to compound treatment may suggest a dependent mechanism of action on the cancer molecular profile. Acteoside activates a strong antioxidant response. A study suggested that acteoside may exert dose-dependent pro-oxidant effects that mobilize Nrf-2 activation. Additionally, inhibiting protein kinase C (PKC) affects the signaling status of many oncogenic pathways; at high concentrations (after parenteral administration), it mobilizes antitumor-reactive immune responses. The in vivo antitumor effect of acteoside depends on the route of administration and the achieved concentration in the tumor [41].

Combined acteoside and 5- fluorouracil (5-FU) treatment led to the arrest of the G1 phase, enhanced apoptosis by altering the Bax, Bcl-2, and p53 protein levels and gene expression, and reduced the PI3K and p-AKT/total AKT ratio in colorectal cancer cell lines Caco-2 and HCT-116. Moreover, the data suggest a potential role of acteoside in a reduction in the resistance of CRC to 5-FU via targeting the PI3K/AKT signaling pathway [42].

Acteoside and plantamajoside have very similar structures (Figure 1 and Figure 2), so the question arises of which differences in the chemical structures of compounds could determine their activity. In early investigations of acteoside, its hydrolytic products (desrhamnosyl acteoside, caffeic acid, methyl caffeate, 3,4-dihydroxyphenylethyl alcohol(=hydroxytyrosol), 3,4-dihydroxyphenethyl glucoside(=dopeol glucoside)), and the same products could be obtained from plantamajoside and related compounds (martynoside; cinnamic, *p*-coumaric, ferulic, and sinapic acids; isomers of dihydroxybenzoic acids) by using the MTT test in murine melanoma (B16F10), human gastric adenocarcinoma (MK-1), and human uterine carcinoma (HeLa) cells. The authors concluded that an *ortho*-dihydroxyphenolic system (3,4- and 3′,4′-dihydroxy) was important for the activity. At the same time, the kind of sugar, glucose, or rhamnose had no influence [36,43,44]. The aglycone common to acteoside and plantamajoside, the 3,4-dihydroxyphenylethyl alcohol named hydroxytyrosol, is abundant in the leaves and fruits of the olive tree (*Olea europaea* L.), and possesses numerous properties that are beneficial to human health, including anticancer activities shown in the breast (MCF-7, MDA-MB-231), liver (HepG2, Huh7), colon (Caco-2, DLD1, LSI80), prostate (LNCaP, C4-2), and glioblastoma (U-87 MG) cancer cell lines while considering the molecular mechanisms of action [45].

Acteoside and plantamajoside differ structurally by one sugar. In our experiments, both compounds decreased cell viability to 30% or less in the cell lines of OVCAR-3, MCF-7, MDA-MB-231, U138-MG, and HepG2; however, acteoside at the lower dose of 200 µM, and plantamajoside at the higher dose of 300 µM. Both compounds showed the weakest activity towards the cell lines of MCF-12A, U251-MG, and Caco-2. These differences in activity imply the influence of terminal sugars rhamnose and glucose.

Plantamajoside showed inhibitory activity on the viability of human cancer cells in the following cell lines: breast MDA-MB-231 [46], hepatocellular carcinoma Huh7 [47,48], PLC/PRF 5, THLE-2 [47] and HepG2 [48,49,50], cervical SiHa and CaSki [51], esophageal Eca-109 and TE-1 [52], and malignant melanoma A2058 [53]. Comparisons with our results are difficult since IC_50_ values were not reported in those investigations. 

The antiproliferative activity of plantamajoside on breast line MDA-MB-231, measured using counting cell kit 8 test (WST-8, a version of the MTT test), showed a 50% decrease in cell viability at the dose of 250 µg/mL (390 µM) after 24 h, and at the dose of 125 µg/mL (195 µM) after 48 h [46]. Those values were close to those found in our investigations: 367 and 312 µM, respectively.

An anticancer agent should be simultaneously nontoxic to normal healthy cells. In our investigations, acteoside and plantamajoside showed similar and noticeably lower cytotoxicity in nontumorigenic cell line MCF-12A compared to that in the studied cancer cells (except Caco-2 and U251-MG). The compounds decreased the cell viability of MCF-12A to less than 30% at the dose of 400 µM, which is much higher than the doses in the cancer cell lines (200 and 300 µM, respectively). Additionally, the 3-week treatment of pre-senescent normal human diploid fibroblasts with acteoside revealed no cytotoxic effect. Moreover, it increased cellular growth and contributed to the slight delay of cellular senescence. Furthermore, oral delivery in Wistar rats alleviated the inflammatory effects of the dermal injection of doxorubicin [41].

Acteoside, when packed in liposomic nanoparticles, was less cytotoxic in normal human lung cells MRC-5 (IC_50_ 24 µM) than it was in cancer glioblastoma cell lines T98G and U-138 MG (2.9 and 4.0 µM, respectively) 54].

Plantamajoside is noncytotoxic in a few normal cell lines, such as Chinese hamster ovaries (CHO) [46], melanocytes [53] and liver L-02 [48]. 

There are several biological effects underlying the anticancer activity of acteoside, and numerous investigations have disclosed the molecular mechanisms responsible for those effects. The main effects are oxidative stress, apoptosis, and antiproliferative, antiangiogenesis, antimetastasis, anti-invasion, antitumor, and anti-inflammatory effects, and synergistic effects with anticancer drugs [24,27]. 

Acteoside activity can be enhanced with a specific formulation. Acteoside in 190 nm liposomal nanoparticles, able to penetrate the membrane in the process of macropinocytosis, showed significantly enhanced activity from IC_50_ of 85.0 and 44.0 µM to IC_50_ 2.9 and 4.0 µM in glioblastoma cell lines T98G and U-138 MG, respectively, through an increase in apoptosis-associated proteins p35 and caspase-3 [54]. Acteoside may also enhance the activity of anticancer agents. A combination treatment of temozolomide and acteoside of the C6 rat glioblastoma cell line at the doses of 5 mM and 50 µM showed no significant effect on cell viability when administered separately (ca. 52% and 80%) and suppressed cell viability to 30%. The underlying effects were apoptosis and autophagy in C6 cells, and the acteoside contribution was oxidative stress and the induction of mitogen-activated protein kinase pathway gene expression [55]. 

Less frequent reports on the anticancer activity of plantamajoside have indicated similar effects to those of acteoside. In human breast cancer cell line MDA-MB-231, plantamajoside inhibited cell proliferation, migration, and invasion by decreasing the activity of matrix metalloproteinase-9 and -2 [39]. In the HepG2 hepatocarcinoma cell line, plantamajoside exerted activity by suppressing the epithelial–mesenchymal transition (EMT) by downregulating the HIF-1α signaling pathway, inhibited cell migration and invasion, and suppressed the in vivo growth and metastasis of implanted tumors in mice [49]. Plantamajoside showed synergism by increasing metformin cytotoxicity in liver cancer cell lines HepG2 and Huh-7, where it suppressed the activation of Akt/GSK3β signaling [48]. The flavonoid triglycoside showed weak cytotoxicity. Isorhamnetin triglycosides (the exact positions of the sugar moieties were not determined) are less active than isorhamnetin diglycosides in colon cancer cells [56]. Isorhamnetin, in contrast to acteoside and plantamajoside, has no free 3′,4′-dihydroxyphenol system due to the methyl group blocking hydroxyl at the 3′ position. Another flavonoid, apigenin (5,7,4′-trihydroxyflavone), without sugar substitution, revealed much more effective antimetastatic activity in human breast cancer line MCF-7 than that of acteoside; both were isolated from *Anisomeles indica* (L.) Kuntze (Lamiaceae) [57]. A *C*-glucosylflavone, orientin, with a free 3′,4′-dihydroxy system showed weaker activity in glioblastoma cell lines T98G and U-138MG, both free (IC_50_ > 100 µM) and nanoformulated in liposomic substance (IC_50_ 12.0 and 19.5 µM), than that of acteoside (free IC_50_ 85 and 44 µM, nanoformulated IC_50_ 2.9 and 4.0 µM) (85) [54]. 

The anticancer activity of *Plantago lanceolata* has been analysed a few times, but no study has related it to the presence of phenylethanoid glycosides. The methanol extract of *P. lanceolata* showed 50% growth-inhibitory values (GI_50%)_ of >250, 47.17 and 50.58 µg/mL in renal adenocarcinoma (TK-10), breast carcinoma (MCF-7) and melanoma (UACC-62) cell lines, respectively. The activity was assigned to the flavonoids luteolin 7-*O*-glucoside and luteolin, and the mechanism of their action was topoisomerase I-mediated DNA damage [8]. The defatted 80% methanolic extract of the herb, when tested in cell lines Hela, MCF-7, HT-29 (colon cancer), and MRC-5 (normal human lung cells), showed IC_50_ values of 172.3, 142.8, 405.5, and 551.7 µg/mL, respectively [17]. A 70% ethanol extract of the leaves exhibited a nonsignificant effect in human breast cell lines MCF-7, MDMAB, and AMJ13, with the exception of CAL51 triple-negative breast cancer cells (IC_50_ 23.7 µg/mL); the inhibition of the viability of normal embryonic fibroblast cells (MEFs) was much weaker (IC_50_ 430 µg/mL) [5]. 

These studies confirmed the significant pharmacological effect of phenylethanoid glycosides in important disease entities. This group of compounds is widespread in about 20 families of higher plants and is widely studied for pharmaceutical, medical, and industrial applications [13,14,58,59]. However, due to the demand for natural medicinal substances, new biotechnological sources of these compounds are also sought [60,61]. In our previous studies, we obtained materials from in vitro cultures of the *Plantago* genus with a high content of acteoside and plantamajoside, e.g., *P. lanceolata*-stabilized callus line with 28.4 mg/g of plantamajoside [62], shoots from micropropagated *P. media* with a 93.0 mg/g acteoside content, the roots of this micropropagated species with a plantamajoside content of 44.1 mg/g [63], and the adventitious roots of *P. ovata* agitated in liquid artificial media producing 33.02 mg/g of acteoside [64]. In terms of the need for these compounds for pharmacological purposes, any new source is valuable. The above studies show that the in vitro cultures of *Plantago* species are a promising system for the biosynthesis of bioactive phenylethanoid glycosides.

## 5. Conclusions

Phenylethanoids are potential compounds with cytotoxic activity against the selected cancer types. For the first time, the activities of acteoside and plantamajoside were compared in one parallel investigation. The two compounds acted on the same cancer cell lines, but acteoside was generally more potent. The difference in one sugar in the chemical structure of these two phenylethanoids influences their cytotoxic effect. Despite the numerous anticancer effects and mechanisms of action found especially for acteoside, the compounds under study appeared to be poorly effective in glioblastoma cell line U251-MG.

## Figures and Tables

**Table 1:** Cytotoxic efficacy of acteoside and plantamajoside against OVCAR-3, MCF-7, MDA-MB-231, MCF-12A, U251-MG, U138-MG, Caco-2, and HepG2 cells, expressed as IC_50_ values. IC_50_ represents the concentration at which a substance exerts half its maximal inhibitory effect. Values that exceeded 500 μM were determined with CompuSyn on the basis of the dose–response curve estimation. Results from three independent experiments are presented (estimations ± SE). Mean values within a row (capital letters) and a column (small letters) within the same letters were not significantly different at *p* < 0.05 using Duncan’s multiple-range test.

	IC_50_ [μM]					
	Acteoside			Plantamajoside		
Cell Line	24 h	48 h	72 h	24 h	48 h	72 h
OVCAR-3	314.1 ± 3.52 ^d,F^	232.0 ± 5.08 ^d,D^	162.8 ± 3.41 ^b,B^	243.5 ± 2.61 ^a,E^	201.3 ± 5.29 ^a,C^	138.9 ± 4.85 ^a,A^
MCF-7	219.1 ± 1.40 ^a,D^	154.2 ± 2.71 ^a,B^	113.1 ± 2.81 ^a,A^	312.9 ± 4.11 ^c,E^	243.8 ± 3.57 ^c,D^	170.8 ± 2.50 ^c,C^
MDA-MB-231	312.2 ± 3.28 ^d,D^	244.9 ± 4.96 ^e,B^	200.2 ± 2.45 ^d,A^	366.7 ± 3.71 ^d,e,E^	311.7 ± 4.83 ^d,D^	263.1 ± 3.20 ^d,C^
MCF-12A	362.3 ± 1.86 ^e,D^	346.8 ± 3.67 ^f,C^	326.3 ± 3.89 ^f,B^	376.3 ± 7.91 ^e,E^	339.2 ± 4.31 ^e,C^	296.2 ± 4.90 ^e,A^
U251-MG	3152.7 ± 4.15 ^f,F^	2412.5 ± 7.97 ^h,E^	1165.3 ± 6.05 ^g,C^	2099.9 ± 5.61 ^g,D^	1117.3 ± 6.21 ^g,B^	985.6 ± 5.71 ^g,A^
U138-MG	274.3 ± 2.61 ^c,D^	201.9 ± 4.90 ^b,B^	156.6 ± 4.74 ^b,A^	359.5 ± 2.75 ^d,F^	342.1 ± 2.35 ^e,E^	266.7 ± 3.88 ^d,C^
Caco-2	507.6 ± 4.05 ^e,F^	466.9 ± 8.71 ^g,E^	280.3 ± 6.13 ^e,A^	447.0 ± 6.16 ^f,D^	378.0 ± 5.23 ^f,C^	316.4 ± 4.27 ^f,B^
HepG2	261.3 ± 3.52 ^b,D^	219.6 ± 2.65 ^c,C^	173.8 ± 1.59 ^c,B^	284.7 ± 5.04 ^b,E^	228.2 ± 1.95 ^b,C^	156.1 ± 2.90 ^b,A^

## Data Availability

Not applicable.
